# Factors associated with smoking cessation among university students, faculty, and staff in Saudi Arabia: A cross-sectional study

**DOI:** 10.18332/tid/231160

**Published:** 2026-10-26

**Authors:** Abdulaziz F. Alshahrani, Abdullah H. Alhalafi, Mubarak F. Alshahrani, Saeed F. Alshahrani, Aws A. Abuliat, Mubarak A. Alqahtani, Mohammed A. Alharthi, Nawaf A. Alsoiry, Mashael O. Alsaadi, Mohammed M. Alshahrani, Raydaa A. Alshomrani, Huthayfah A. Alghamdi

**Affiliations:** 1 Department of Family and Community Medicine, College of Medicine, University of Bisha, Bisha, Saudi Arabia; 2 Department of Emergency Medicine, Security Forces Hospital, Riyadh, Saudi Arabia; 3 Medicine and Surgery Department, King Khalid University, Saudi Board of Family Medicine, Abha, Saudi Arabia

**Keywords:** smoking cessation, tobacco use, associated factors, university population, Saudi Arabia

## Abstract

**INTRODUCTION:**

Smoking cessation is an important public health priority given the substantial health risks associated with tobacco use. This study aimed to assess factors associated with former-smoker status among university students, faculty members, and employees in Saudi Arabia.

**METHODS:**

A cross-sectional study was conducted at the University of Bisha, Saudi Arabia, from August 2024 to April 2025. Eligible participants were adults affiliated with the university who self-identified as current or former smokers. Data were collected using a self-administered online questionnaire. Associations between participant characteristics and former-smoker status were assessed using Pearson’s chi-squared test, followed by univariable and multivariable logistic regression. Because only 48 outcome events were available, the multivariable model was restricted to four conceptually selected variables, with all categorical predictors entered as indicator variables.

**RESULTS:**

Among 533 respondents, 113 were current or former smokers; 48 (42.5%) were former smokers and 65 (57.5%) were current smokers. Overall, 83 participants (73.5%) were classified as having quit or attempted to quit smoking. Being classified as having quit or attempted to quit was associated with female sex (p=0.030), non-daily smoking (p=0.009), and cigarettes per day (p=0.023). In univariable analyses, female sex (OR=4.14; 95% CI: 1.54–11.12; p=0.005) and non-daily smoking (OR=6.58; 95% CI: 2.22–19.52; p=0.001) were associated with former-smoker status. In the adjusted analysis, non-daily smoking remained the only independent association (AOR=5.47; 95% CI: 1.60–18.68; p=0.007). The association with female sex was attenuated and no longer statistically significant (AOR=3.01; 95% CI: 0.97–9.36; p=0.057), whereas neither age nor any cigarettes per day category remained independently associated with former-smoker status.

**CONCLUSIONS:**

Non-daily smoking was the only factor independently associated with former-smoker status. These findings are exploratory and limited by the single-university convenience sample and the imprecision of some estimates. Larger longitudinal studies using validated measures of tobacco exposure and sustained abstinence are needed to confirm these associations.

## INTRODUCTION

Tobacco use is responsible for about 5 million deaths among adults aged above 30 years globally, representing approximately 12% of all deaths in that age group^1^. These figures make tobacco use one of the leading causes of preventable mortality worldwide, as it is a major risk factor for several non-communicable diseases, including cardiovascular disease, lung cancer, and chronic respiratory diseases^2^. According to Dai et al.^3^, between 0.94 and 1.47 billion people regularly smoke tobacco globally, with smoking prevalence estimated at 32.6% among men and 6.5% among women. In Saudi Arabia, a 2024 national cross-sectional survey of 457 medical residents reported that 33.5% were current smokers^4^. Among the general population, a 2018 study covering all 13 administrative regions of Saudi Arabia reported a smoking prevalence of 21.4%, with a prevalence of 32.5% among males and 3.9% among females^5^.

Previous studies have identified a range of individual and external factors associated with successful smoking cessation^6,7^. Smoking cessation is a dynamic and difficult process for many individuals, often requiring multiple quit attempts before achieving long-term abstinence^8^. The factors involved are multifaceted and include individual-level characteristics such as age, gender, education level, nicotine dependence, and motivation to quit, as well as interpersonal influences such as family support and peer pressure^8^. Factors reported in previous studies include low nicotine dependence, male sex, higher education, being married, consuming fewer cigarettes per day, and having fewer smokers at home^8^.

Saudi experts have identified smoking relapse and factors associated with long-term smoking cessation as important national tobacco-research priorities^9^. Despite this, little information is available on factors associated with former-smoker status among university populations in Saudi Arabia. University communities are of particular interest because they include young adults at the age at which tobacco use is typically initiated, alongside older faculty and staff with longer smoking histories. Although students, faculty members, and employees differ in age, socioeconomic background, and occupational status, they share the same institutional tobacco policies, health-promotion activities, and physical environment on the university premises. These three groups were therefore examined together, while occupation and income were evaluated as potential correlates and reported separately in the analysis. Examining this population may provide information relevant to the development of tobacco-control strategies in university settings.

## METHODS

### Study design and population

This cross-sectional study was conducted at the University of Bisha, Saudi Arabia, between August 2024 and April 2025 among students, faculty members, and employees. At the time of the study, the university population was estimated at approximately 19065 individuals, including about 15000 students and 4000 faculty members and staff. Eligible participants were those aged ≥18 years, affiliated with the university, resident in the city where the university is located during the data-collection period, and self-identified as current or former smokers. Smoking status was determined from self-reported questionnaire responses. Participants were asked whether they had smoked at least 100 combustible cigarettes during their lifetime and whether they currently smoked; this threshold was applied to cigarette smokers in line with standard epidemiological definitions. Participants who primarily used waterpipe or electronic cigarettes were classified according to their self-reported current or former tobacco-use status, which is acknowledged as a limitation. Participants were excluded if they were younger than 18 years, not affiliated with the university, non-smokers, or submitted incomplete responses.

### Data collection tools

Data were collected using a self-administered online questionnaire (Supplementary file) adapted from previously published literature and clinical guidelines on tobacco use and smoking cessation^10,11^. The questionnaire contained approximately 25 items across four sections: sociodemographic characteristics; smoking behaviors (type and frequency of smoking); perceived reasons for smoking initiation and cessation; and cessation history, including time since the most recent cessation attempt, type of support received, pharmacological treatment, and number of previous quit attempts. The survey took approximately 5–7 minutes to complete. It was distributed to all university colleges and departments through official email, and two reminders were sent at two-week intervals through institutional groups on platforms such as WhatsApp and Telegram. For analysis, responses indicating psychotherapy or behavioral support were categorized as counseling; participants reporting pharmacological treatment only were categorized as pharmacotherapy; and participants reporting both were categorized as combined treatment.

### Variables and coding

The primary outcome variable was former-smoker status, defined as being a former smoker (coded 1) compared with a current smoker (coded 0); this was the outcome modeled in the multivariable logistic regression. A secondary descriptive outcome, being classified as having quit or attempted to quit smoking at least once (yes, no), was examined in bivariate analysis only and is reported separately. This classification included participants who answered the quit/attempt item affirmatively as well as former smokers classified as having quit on the basis of their smoking status. This outcome is distinct from former-smoker status, and the number of participants meeting each differs.

Age was analyzed as a continuous variable (odds ratio per one-year increase). Nationality was categorized as Saudi or non-Saudi and was included in the descriptive and bivariate analyses. Variables examined in the regression analyses were analyzed as categorical variables with the following categories and reference groups: gender (male [ref.], female); occupation (student [ref.], administration employee, faculty member, security); marital status (single [ref.], married, divorced); monthly income in Saudi Riyals (<3000 [ref.], 3000–5999, 6000–10000, >10000); type of tobacco used (cigarettes [ref.], chewing tobacco, electronic cigarettes, waterpipe); daily smoking (yes [ref.], no); and cigarettes per day (≤10 [ref.], 11–20, 21–30, >30).

Type of tobacco used was recorded as a multiple-response item listing cigarettes, waterpipe, electronic cigarettes, chewing tobacco and tobacco bags; 57 of the 113 participants selected more than one product. For analysis, each participant was assigned a single primary product, taken as the first product listed in the questionnaire, and tobacco bags were grouped with chewing tobacco because of the small number of users. Each multi-category variable was entered as a set of indicator (dummy) variables rather than as an ordinal score, so that a separate odds ratio was estimated for each category relative to its reference group. Cigarettes per day was modeled as a four-level categorical variable and not as an ordinal score.

### Sampling and sample size

A convenience sampling approach was used to recruit eligible participants. The minimum required sample size was calculated using Cochran’s formula. As no precise prior estimate was available for the target population, an expected prevalence of 50%, a 5% margin of error, and a 95% confidence level were applied, yielding a minimum required sample size of 385 participants^12^. The questionnaire was completed by 533 respondents, of whom 113 met the eligibility criterion of current or former smoking; the analytical sample was therefore smaller than the calculated minimum, which is acknowledged as a limitation.

### Statistical analysis

Data were initially entered into Microsoft Excel for cleaning, which included removal of incomplete responses and duplicates. Duplicate responses were identified on the basis of identical timestamps, matching demographic characteristics such as age and gender, and repeated response patterns; where duplicates were identified, only the first complete response was retained. Cleaned data were then imported into IBM SPSS Statistics version 29. Descriptive statistics are presented as frequencies and percentages for categorical variables and as mean ± standard deviation (SD) for continuous variables. Associations between categorical variables were assessed using Pearson’s chi-squared test. All tests were two-sided. Type of cessation support and time since support were not tested against this outcome because only participants who had quit or attempted could have received support, which would render such comparisons circular. Unadjusted (crude) odds ratios (ORs) were estimated for every variable using separate univariable logistic regression models. Multivariable logistic regression was then performed to identify factors independently associated with former-smoker status, using the coding and reference categories described above. Because the analytical sample contained only 48 outcome events, a model containing all eight candidate variables would have required 17 estimated parameters, corresponding to approximately 2.8 events per estimated parameter, well below the conventional minimum of ten. The variables retained in the multivariable model were therefore specified in advance on conceptual rather than statistical grounds: age and gender were retained as core demographic confounders, and daily smoking and cigarettes per day were retained as the tobacco-exposure variables central to the study question. No stepwise or significance-based selection procedure was used, and no variable was retained or removed on the basis of its p-value. The resulting model contained six estimated parameters, corresponding to 8.0 events per parameter. Model calibration was assessed using the Hosmer-Lemeshow test, and the stability of the estimates was examined in sensitivity analyses in which each omitted variable was added to the model in turn and in which the full eight-variable model was refitted. Results are presented as crude odds ratios (ORs) and adjusted odds ratios (AORs) with 95% confidence intervals (CIs). A p<0.05 was considered statistically significant.

### Ethical considerations

Ethical approval for this study was obtained from the Local Permanent Committee of Research Bioethics, College of Medicine, University of Bisha, Saudi Arabia (Approval No. UB-RELOC H-06-BH-087/[1802.24], dated 21 August 2024). Electronic informed consent was obtained from all participants before participation, and responses were collected anonymously.

## RESULTS

Of the 533 respondents who completed the questionnaire, 113 (21.2%) were current or former smokers and were included in the analysis. Most participants were male (90; 79.6%), Saudi (106; 93.8%), students (92; 81.4%), single (95; 84.1%), and earning <3000 Saudi Riyals (SAR) per month (82; 72.6%). The mean age was 24.55 ± 7.96 years. Sixty-five participants (57.5%) were current smokers and 48 (42.5%) were former smokers. The most commonly used tobacco products were cigarettes (73; 64.6%) and electronic cigarettes (28; 24.8%). Most participants smoked daily (91; 80.5%), and more than half reported smoking ≤10 cigarettes per day (61; 54.0%) (Table 1).

**Table 1:** Sociodemographic and smoking characteristics of current and former smokers in a cross-sectional study at the University of Bisha, Saudi Arabia, August 2024–April 2025 (N=113)

*Variable*	*Category*	*n (%)*
**Age** (years), mean ± SD		24.55 ± 7.96
**Gender**	Male (ref.)	90 (79.6)
	Female	23 (20.4)
**Occupation**	Student (ref.)	92 (81.4)
	Administration employee	5 (4.4)
	Faculty member	12 (10.6)
	Security	4 (3.5)
**Nationality**	Saudi	106 (93.8)
	Non-Saudi	7 (6.2)
**Marital status**	Single (ref.)	95 (84.1)
	Married	14 (12.4)
	Divorced	4 (3.5)
**Monthly income (SAR)**	<3000 (ref.)	82 (72.6)
	3000–5999	7 (6.2)
	6000–10000	12 (10.6)
	>10000	12 (10.6)
**Smoking status**	Current smoker	65 (57.5)
	Former smoker	48 (42.5)
**Type of tobacco used**	Cigarettes (ref.)	73 (64.6)
	Chewing tobacco	3 (2.7)
	Electronic cigarettes	28 (24.8)
	Waterpipe	9 (8.0)
**Daily smoking**	Yes (ref.)	91 (80.5)
	No	22 (19.5)
**Cigarettes per day**	≤10 (ref.)	61 (54.0)
	11–20	39 (34.5)
	21–30	7 (6.2)
	>30	6 (5.3)

SAR: Saudi Riyals. SD: standard deviation. ref.: reference category used in the multivariable logistic regression model (Table 4). Percentages may not sum to 100% due to rounding.

Perceived factors and cessation practices are presented in Table 2. Among the 113 participants, the most commonly reported perceived reasons for smoking initiation were smoking for fun (52/113; 46.0%) and peer pressure or having friends who smoked (41/113; 36.3%). Health concerns were the most frequently reported perceived reason for cessation (52/113; 46.0%), followed by peer pressure (39/113; 34.5%); religious beliefs (7/113; 6.2%) and cost (7/113; 6.2%) were reported less frequently. Among the 65 current smokers, 43 (66.2%) expressed a desire to quit smoking, whereas 22 (33.8%) did not; among these 22, the most commonly reported barriers were fear of mood changes (17/22; 77.3%) and previous failed attempts (4/22; 18.2%). Overall, 83 of the 113 participants (73.5%) were classified as having quit smoking or made at least one quit attempt. Of these 83 participants, 81 answered the quit/attempt item affirmatively; the remaining two were former smokers who answered the item negatively and were classified as having quit on the basis of their smoking status. Among the 81 participants who answered the quit/attempt item affirmatively, 27 (33.3%) reported one quit attempt, 15 (18.5%) reported two attempts, 17 (21.0%) reported three attempts, 7 (8.6%) reported four attempts, and 15 (18.5%) reported five or more attempts. All 81 reported the form of cessation support received: counseling alone was the most common (61/81; 75.3%), followed by combined counseling and pharmacotherapy (14/81; 17.3%) and pharmacotherapy alone (6/81; 7.4%). Among the 20 participants who received any pharmacotherapy, nicotine replacement therapy was the most frequently reported medication (8/20; 40.0%).

**Table 2:** Perceived factors associated with smoking initiation and cessation, and cessation practices, among current and former smokers in a cross-sectional study at the University of Bisha, Saudi Arabia, August 2024–April 2025 (N=113)

*Item*	*Category*	*n (%)*
**Perceived factors**		
**Perceived reasons for smoking initiation**	Smoking for fun	52 (46.0)
	Peer pressure or friends who smoke	41 (36.3)
	Stress	11 (9.7)
	Social imitation	4 (3.5)
	Significant life event	4 (3.5)
	Family member	1 (0.9)
**Perceived factors affecting smoking cessation**	Health concerns	52 (46.0)
	Peer pressure	39 (34.5)
	Religious beliefs	7 (6.2)
	To save money/cost of cigarettes	7 (6.2)
	Familial pressure or respect for family values	3 (2.7)
	Side effects of smoking heard or observed	3 (2.7)
	Legal prohibitions	1 (0.9)
	To have a better life	1 (0.9)
**Desire to quit by current smokers** (N=65)		
**Desire to quit smoking**	Yes	43 (66.2)
	No	22 (33.8)
**Perceived reasons for unwillingness to quit** (N=22)	Fear of mood changes	17 (77.3)
	Previous failed attempt(s)	4 (18.2)
	Fear of failure	1 (4.5)
**Cessation practices**		
**Classified as having quit or attempted to quit smoking**	Yes	83 (73.5)
	No	30 (26.5)
**Number of quit attempts among participants who answered the quit/attempt item affirmatively** (N=81)	1	27 (33.3)
	2	15 (18.5)
	3	17 (21.0)
	4	7 (8.6)
	≥5	15 (18.5)
**Time since most recent cessation support** (N=81)	≤6 months	69 (85.2)
	1 year	3 (3.7)
	2 years	6 (7.4)
	>2 years	3 (3.7)
**Type of cessation support received** (N=81)	Counselling only	61 (75.3)
	Combined counselling and pharmacotherapy	14 (17.3)
	Pharmacotherapy only	6 (7.4)
**Medication received by participants receiving any pharmacotherapy** (N=20)	Nicotine replacement therapy	8 (40.0)
	Varenicline	1 (5.0)
	Combined pharmacotherapy	1 (5.0)
	Other	10 (50.0)

Denominators differ between items and are stated for each; percentages are calculated within the stated denominator. Of the 83 participants classified as having quit or attempted to quit smoking, 81 answered the quit/attempt item affirmatively. The remaining two were former smokers who answered negatively to this item and had no subsequent quit-attempt or cessation-support data. Therefore, analyses of the number of quit attempts and cessation support were based on the 81 participants with affirmative responses.

With regard to the distribution of participants classified as having quit or attempted to quit smoking, 83 of 113 (73.5%) were classified as having done so, while 30 (26.5%) were not. In the bivariate analysis, being classified as having quit or attempted to quit smoking was significantly associated with gender (p=0.030), daily smoking status (p=0.009), and cigarettes per day (p=0.023). Classification as having quit or attempted to quit applied to 21 of 23 female participants (91.3%) compared with 62 of 90 male participants (68.9%), and to 21 of 22 non-daily smokers (95.5%) compared with 62 of 91 daily smokers (68.1%). By consumption of cigarettes per day: 50 of 61 participants smoked ≤10 (82.0%), 25 of 39 smoked 11–20 (64.1%), 6 of 7 smoked 21–30 (85.7%), and 2 of 6 smoked >30 (33.3%). Occupation (p=0.091), nationality (p=0.101), marital status (p=0.054), monthly income (p=0.096), and type of tobacco used (p=0.566) were not significantly associated with this outcome. Several categories contained no participants in one group, and the corresponding p-values should therefore be interpreted with caution (Table 3).

**Table 3:** Association between participant characteristics and being classified as having quit or attempted to quit smoking among current and former smokers in a cross-sectional study at the University of Bisha, Saudi Arabia, August 2024–April 2025 (N=113)

*Variable*	*Category*	*Quit or attempted n (%)*	*Neither n (%)*	*p*
**Gender**	Male (ref.)	62 (68.9)	28 (31.1)	0.030*
	Female	21 (91.3)	2 (8.7)	
**Occupation**	Student (ref.)	63 (68.5)	29 (31.5)	0.091
	Administration employee	5 (100.0)	0 (0.0)	
	Faculty member	11 (91.7)	1 (8.3)	
	Security	4 (100.0)	0 (0.0)	
**Nationality**	Saudi	76 (71.7)	30 (28.3)	0.101
	Non-Saudi	7 (100.0)	0 (0.0)	
**Marital status**	Single (ref.)	66 (69.5)	29 (30.5)	0.054
	Married	14 (100.0)	0 (0.0)	
	Divorced	3 (75.0)	1 (25.0)	
**Monthly income** (SAR)	<3000 (ref.)	55 (67.1)	27 (32.9)	0.096
	3000–5999	6 (85.7)	1 (14.3)	
	6000–10000	11 (91.7)	1 (8.3)	
	>10000	11 (91.7)	1 (8.3)	
**Type of tobacco used**	Cigarettes (ref.)	51 (69.9)	22 (30.1)	0.566
	Chewing tobacco	2 (66.7)	1 (33.3)	
	Electronic cigarettes	22 (78.6)	6 (21.4)	
	Waterpipe	8 (88.9)	1 (11.1)	
**Daily smoking**	Yes (ref.)	62 (68.1)	29 (31.9)	0.009*
	No	21 (95.5)	1 (4.5)	
**Cigarettes per day**	≤10 (ref.)	50 (82.0)	11 (18.0)	0.023*
	11–20	25 (64.1)	14 (35.9)	
	21–30	6 (85.7)	1 (14.3)	
	>30	2 (33.3)	4 (66.7)	

Percentages are row percentages within each category. The outcome in this table is being classified as having quit or attempted to quit smoking (83/113; 73.5%), which is distinct from former smoker (48/113; 42.5%) modeled in Table 4. Associations were assessed using the chi-squared test or Fisher’s exact test, as appropriate; all tests were two-tailed. Several categories contain no participants in one column, and the corresponding p-values should be interpreted with caution. Type of cessation support and time since support were not tested here because only participants who had quit or attempted could have received support. SAR: Saudi Riyals.

* Statistically significant at p<0.05.

The remaining analyses concern former-smoker status, the outcome modeled in the multivariable analysis, which was present in 48 of the 113 participants (42.5%).

In the univariable analyses, female sex (OR=4.14; 95% CI: 1.54–11.12; p=0.005), married status (OR=3.92; 95% CI: 1.14–13.42; p=0.030) and non-daily smoking (OR=6.58; 95% CI: 2.22–19.52; p=0.001) were associated with former-smoker status, whereas age, occupation, monthly income, type of tobacco used and cigarettes per day were not. None of the three participants who primarily used chewing tobacco was a former smoker, so no odds ratio could be estimated for that category. In the multivariable model, participants who did not smoke daily had higher odds of former-smoker status than daily smokers (AOR=5.47; 95% CI: 1.60–18.68; p=0.007). The association with female sex was attenuated after adjustment and did not reach statistical significance (AOR=3.01; 95% CI: 0.97–9.36; p=0.057). Neither age (AOR=1.03 per one-year increase; 95% CI: 0.98–1.09; p=0.206) nor any category of cigarettes per day was independently associated with former-smoker status (11–20 cigarettes/day, AOR=1.41; 95% CI: 0.55–3.62; 21–30 cigarettes/day, AOR=2.06; 95% CI: 0.39–10.96; >30 cigarettes/day, AOR=0.55; 95% CI: 0.06–5.36). The model was adequately calibrated (Hosmer-Lemeshow χ^2^=8.75, df=8, p=0.363), and the association with non-daily smoking was consistent across all sensitivity specifications examined (AOR=4.76–5.72; all p<0.03) (Table 4).

**Table 4:** Unadjusted and adjusted logistic regression analysis of factors associated with former-smoker status among current and former smokers in a cross-sectional study at the University of Bisha, Saudi Arabia, August 2024–April 2025 (N=113; 48 former smokers, 65 current smokers)

*Variable*	*Category*	*Former smokers n/N (%)*	*OR (95% CI)*	*p*	*AOR (95% CI)*	*p*
**Age** (years)	Per one-year increase	48/113 (42.5)	1.02 (0.97–1.07)	0.494	1.03 (0.98–1.09)	0.206
**Gender**	Male (ref.)	32/90 (35.6)	1.00		1.00	
	Female	16/23 (69.6)	4.14 (1.54–11.12)	0.005*	3.01 (0.97–9.36)	0.057
**Occupation**	Student (ref.)	35/92 (38.0)	1.00		NI	
	Administration employee	4/5 (80.0)	6.51 (0.70–60.66)	0.100	NI	
	Faculty member	8/12 (66.7)	3.26 (0.91–11.62)	0.069	NI	
	Security	1/4 (25.0)	0.54 (0.05–5.43)	0.603	NI	
**Marital status**	Single (ref.)	37/95 (38.9)	1.00		NI	
	Married	10/14 (71.4)	3.92 (1.14–13.42)	0.030*	NI	
	Divorced	1/4 (25.0)	0.52 (0.05–5.21)	0.580	NI	
**Monthly income** (SAR)	<3000 (ref.)	30/82 (36.6)	1.00		NI	
	3000–5999	3/7 (42.9)	1.30 (0.27–6.20)	0.742	NI	
	6000–10000	7/12 (58.3)	2.43 (0.71–8.32)	0.159	NI	
	>10000	8/12 (66.7)	3.47 (0.96–12.49)	0.057	NI	
**Type of tobacco used**	Cigarettes (ref.)	30/73 (41.1)	1.00		NI	
	Chewing tobacco	0/3 (0.0)	NE^†^		NI	
	Electronic cigarettes	12/28 (42.9)	1.08 (0.45–2.60)^‡^	0.872	NI	
	Waterpipe	6/9 (66.7)	2.87 (0.66–12.37)^‡^	0.158	NI	
**Daily smoking**	Yes (ref.)	31/91 (34.1)	1.00		1.00	
	No	17/22 (77.3)	6.58 (2.22–19.52)	0.001*	5.47 (1.60–18.68)	0.007*
**Cigarettes per day**	≤10 (ref.)	29/61 (47.5)	1.00		1.00	
	11–20	15/39 (38.5)	0.69 (0.30–1.56)	0.373	1.41 (0.55–3.62)	0.477
	21–30^§^	3/7 (42.9)	0.83 (0.17–4.01)	0.814	2.06 (0.39–10.96)	0.397
	>30^§^	1/6 (16.7)	0.22 (0.02–2.00)	0.179	0.55 (0.06–5.36)	0.610

AOR: adjusted odds ratio. CI: confidence interval.

† Not estimable: none of the three participants who primarily used chewing tobacco was a former smoker (complete separation), so no finite odds ratio exists.

‡ Estimated in a univariable model excluding the three chewing-tobacco users (n=110), since their inclusion prevented model convergence.

§ Category contains fewer than 10 participants; the corresponding estimates are imprecise and should be interpreted with caution. SAR: Saudi Riyals. NI: not included in the multivariable model. NE: not estimable. Statistically significant at p<0.05.

## DISCUSSION

In this cross-sectional study of university students, faculty members, and employees who reported current or former tobacco use, non-daily smoking was independently associated with higher odds of former-smoker status. Female sex showed a comparable association in unadjusted analysis that was attenuated after adjustment, and cigarettes per day was not independently associated with the outcome. Most participants were classified as having quit or attempted to quit at least once, and in bivariate analysis this was more frequent among women, non-daily smokers, and those with fewer cigarettes per day. Smoking for fun and peer influence were the most frequently reported perceived reasons for initiation, and health concerns were the most frequently reported perceived reason for cessation. Counseling was the most frequently reported form of cessation support. These findings are exploratory and describe associations observed at a single point in time.

Non-daily smoking was associated with higher odds of former-smoker status and was the only association that persisted after adjustment and across all sensitivity analyses. Cigarettes per day, by contrast, showed no independent association with former-smoker status in either unadjusted or adjusted analysis, and the two highest categories each contained fewer than ten participants. These observations are broadly consistent with previous evidence suggesting that lower levels of tobacco exposure and nicotine dependence are associated with more favorable cessation-related outcomes^11,13,14^. The present data, however, support this only for the distinction between daily and non-daily smoking and do not demonstrate a graded relationship across cigarette-consumption categories; given the imprecision of the estimates, these findings should be regarded as hypothesis-generating.

The proportion of participants who reported having quit or attempted to quit is broadly comparable with reports from other settings. The Centers for Disease Control and Prevention (CDC) has reported that quit attempts are common among adult cigarette smokers in the United States^15^. The most commonly reported perceived reasons for smoking initiation in the present study were fun and peer pressure, particularly having friends who smoked. This is consistent with the findings of Anjum et al.^10^ and Al-Nimr et al.^16^, who identified peer influence and social factors related to smoking initiation among young people.

Approximately two-thirds of the current smokers in this study reported a desire to quit, and health concerns were the most frequently reported perceived reason for cessation. This is consistent with Al-Nimr et al.^16^, who identified health concerns as the predominant reported reason for willingness to quit among Saudi women attending smoking cessation clinics. Among participants who did not wish to quit, fear of mood changes and previous failed attempts were the most commonly reported barriers. Al-Nimr et al.^16^ similarly identified fear of mood changes as an important reported reason for unwillingness to quit among Saudi women attending smoking cessation clinics.

Religious beliefs were reported as a perceived cessation factor by a small proportion of participants in this study. Although the frequency was low, culturally specific occasions may be relevant to cessation behavior in this setting. Monshi et al.^17^ reported that considering quitting during Ramadan, Eids, and birthdays was associated with a greater desire to quit and a higher likelihood of previous quit attempts among Saudi tobacco users. The low frequency observed here may reflect the wording and response options of the questionnaire rather than the absence of such influences, and this warrants examination in studies designed specifically to capture culturally meaningful cessation occasions.

Counseling was the most frequently reported form of cessation support in this study, followed by combined counseling and pharmacotherapy and by pharmacotherapy alone; nicotine replacement therapy was the most frequently reported medication. Because participation in, exposure to, and effectiveness of cessation services were not measured, no inference can be drawn about whether any of these forms of support contributed to cessation. National data indicate a substantial gap between awareness and use of cessation services in Saudi Arabia: analysis of the 2019 Global Adult Tobacco Survey found that while 60% of tobacco users were aware of fixed smoking-cessation clinics, only 9% reported having visited one^18^. Whether a comparable gap exists in university settings, and what factors might explain it, remains to be established and would require studies that directly measure awareness, access, and use of cessation services in this population.

### Limitations

Several limitations should be considered when interpreting these findings. First, the cross-sectional design permits the identification of associations but does not allow causal inference or the establishment of temporality, and reverse causality cannot be excluded. Second, the study relied on self-reported data collected through an online questionnaire, which may have introduced recall and information bias, particularly regarding perceived reasons for smoking initiation and cessation history, and may have resulted in misclassification of smoking status, cigarettes per day, and cessation history. Third, the conventional lifetime threshold of 100 cigarettes was applied to cigarette smokers and may not adequately capture lifetime exposure among participants who primarily used electronic cigarettes or waterpipe, which may have led to misclassification within these subgroups.

Fourth, the analytical sample of 113 participants was smaller than the calculated minimum of 385, and recruitment relied on convenience sampling with a low response rate; both limit statistical power and generalizability and may have introduced selection bias. Fifth, the analytical sample contained only 48 participants with the outcome of interest, and several categories contained very few or no participants. A model containing all eight candidate variables would have yielded approximately 2.8 events per estimated parameter and produced unstable estimates; the multivariable model was therefore restricted to four conceptually specified variables, and the estimates for the remaining variables are reported as unadjusted associations only. Even within the reduced model, the estimates remain imprecise, as reflected in the width of the confidence intervals (for example, AOR=5.47; 95% CI: 1.60–18.68 for non-daily smoking), and should not be interpreted as precise measures of effect size. In addition, none of the three participants who primarily used chewing tobacco was a former smoker, so no odds ratio could be estimated for that category, and the two highest cigarettes per day categories contained fewer than ten participants each. Sixth, former-smoker status was relatively common in the analytical sample (42.5%). When an outcome is common, odds ratios diverge from prevalence ratios and may overstate the magnitude of the underlying association; the adjusted odds ratios reported here should therefore be interpreted as measures of association on the odds scale rather than as approximations of relative prevalence. Alternative approaches such as modified Poisson regression with robust standard errors can yield adjusted prevalence ratios, but were not applied here because of the small sample size and sparse category counts, which would be likely to produce unstable estimates. Seventh, the adapted questionnaire was based on previously published literature and clinical guidelines but was not formally validated in this population, and no validated measure of nicotine dependence was administered. Residual confounding from unmeasured factors, including nicotine dependence, mental health status, and social support, may therefore have influenced the findings. In addition, two related but distinct outcomes are reported: having quit or attempted to quit at least once, examined descriptively in bivariate analysis, and former-smoker status, modeled in the multivariable analysis. Only the latter reflects achieved cessation, and findings for one outcome should not be read as applying to the other. Finally, as the study was conducted at a single university in Saudi Arabia, the findings may not be generalizable to other universities or to the wider population.

## CONCLUSIONS

Exploratory cross-sectional associations were identified between former-smoker status and sex and daily smoking status among university students, faculty members, and employees who reported current or former tobacco use. In the adjusted analysis, non-daily smoking was the only factor independently associated with higher odds of former-smoker status. Female sex showed a similar association that did not reach statistical significance after adjustment, and cigarette per day was not independently associated with the outcome. Smoking for fun and peer influence were the most frequently reported perceived reasons for initiation, and health concerns the most frequently reported perceived reason for cessation. These associations were identified in a convenience sample from a single university, were estimated with considerable imprecision, and should be interpreted as exploratory rather than causal. Confirmation requires larger longitudinal studies across multiple institutions using validated definitions of tobacco exposure and sustained abstinence.

## Supplementary Material



## Data Availability

The data supporting this research are available from the authors on reasonable request.
