# Supplementary material for: Factors associated with smoking cessation among university students, faculty, and staff in Saudi Arabia: A cross-sectional study
Source: Tob Induc Dis. 2026 Oct 26;24:10.18332/tid/231160. doi: 10.18332/tid/231160 (PMC13712268; doi:10.18332/tid/231160)
Supplement: Supplementary file 1 [file TID-24-198-s1.pdf]

Tobacco Induced Diseases

Supplementary file

© 2026 Alshahrani A.F. et al.

DOI:
10.18332/tid/231160

The content has been provided by the author(s) and has not been reviewed, verified, or endorsed by European Publishing. It may not have undergone peer review. The views, opinions, and recommendations expressed are solely those of the author(s) and do not necessarily reflect the position of European Publishing. European Publishing accepts no responsibility or liability for any consequences arising from the use of, or reliance on, this content.

Supplementary Materials:

Questionnaires

❖ **Sociodemographic characteristics:**
• **Age:**<br>………………………………………
• **Gender:**<br>❑ Male<br>❑ Female
• **Occupation:**<br>❑ Faculty member<br>❑ Administrative employee<br>❑ Student<br>❑ Security<br>❑ others...
• **Nationality:**<br>❑ Saudi<br>❑ Non-Saudi
• **Marital status:**<br>❑ Single<br>❑ Married<br>❑ Divorced<br>❑ Widowed
• **Monthly income in Saudi Riyals?**<br>❑ None<br>❑ < 3,000<br>❑ 3,000–5,999<br>❑ 6,000–10,000<br>❑ > 10,000<br>❑ Unknown
• **Do you currently smoke?**<br>❑ Yes<br>❑ No
• **Type of smoking? (more than one choice is available)**<br>❑ Cigarettes<br>❑ waterpipe<br>❑ electronic cigarettes<br>❑ Chewing tobacco
• **Daily smoking?**<br>❑ Yes<br>❑ No

- **Number of smoked per day (cigarettes)?**

- [ ] ≤10
- [ ] 11-20
- [ ] 21-30
- [ ] >30

❖ Intervention

- **Smoking abstinence**

- [ ] yes
- [ ] No

- **Period from intervention**

- [ ] 6 months
- [ ] 1 year
- [ ] 2 years

- **Type of intervention** **(more than one choice is available)**

- [ ] Psychotherapy
- [ ] Pharmacotherapy

- **Prescribed medications**

- [ ] Nicotine replacement therapy
- [ ] Varenicline
- [ ] Combined

- **Number of quit attempts**

- [ ] No quit attempts
- [ ] 1
- [ ] 2
- [ ] 3
- [ ] 4
- [ ] ≥ 5

❖ Determinants

- **In your opinion what is the Reasons for smoking initiation? (more than one choice is available)**

- [ ] Peer pressure or Friends who smoke.
- [ ] Smoking as fun
- [ ] Unpleasant event in life
- [ ] Smoking establishes friendships.
- [ ] Family members
- [ ] Stress
- [ ] Social imitation
- [ ] Advertising
- [ ] Other

- **In your opinion what is Factor affecting smoking cessation? (more than one choice is available)**

- [ ] Health concerns
- [ ] Familial pressure or Respect to family values
- [ ] Religious beliefs
- [ ] To save money , Cost of cigarette
- [ ] To have a better life

- ❑ Side effect of smoking heard or observed.
- ❑ Healthy lifestyle advantage
- ❑ Physician advice
- ❑ Future outcomes
- ❑ Legal prohibitions
- ❑ Side effects on others (second hand smokers)
- ❑ Bad smell
- ❑ Antipathy of overuse and length of cigarette use
- ❑ Peer pressure
- ❑ other

- **Do you have a desire to quit smoking**

❑ **Yes**
❑ **No**

- **In your opinion what is Reasons for unwillingness to quit smoking?**

(more than one choice is available)

- ❑ Fear of mood changes
- ❑ Peer pressure
- ❑ Cost of treatment
- ❑ Fear of failure
- ❑ Failed previous attempt(s)
- ❑ Other

- **Have you quit or tried to quit smoking?**

❑ **Yes**
❑ **No**
